# Systematic Review of Hepatitis E Virus in Brazil: A One-Health Approach of the Human-Animal-Environment Triad

**DOI:** 10.3390/ani11082290

**Published:** 2021-08-03

**Authors:** Danny Franciele da Silva Dias Moraes, João R. Mesquita, Valéria Dutra, Maria São José Nascimento

**Affiliations:** 1 Faculty of Veterinary Medicine, Federal University of Mato Grosso, Cuiabá 78060-900, Brazil; dannyfsdm@gmail.com (D.F.d.S.D.M.); valdutra@ufmt.br (V.D.); 2 Secretaria de Estado do Meio Ambiente de Mato Grosso (SEMA), Cuiabá 78050-970, Brazil; 3 Abel Salazar Institute of Biomedical Sciences (ICBAS), University of Porto, 4050-313 Porto, Portugal; 4 Epidemiology Research Unit (EPIUnit), Instituto de Saúde Pública da Universidade do Porto, 4050-600 Porto, Portugal; 5 Faculty of Pharmacy, University of Porto (FFUP), 4050-313 Porto, Portugal; saojose@ff.up.pt

**Keywords:** Brazil, HEV, zoonotic, One Health

## Abstract

**Simple Summary:**

Hepatitis E virus (HEV) is an important causative agent of acute and chronic hepatitis worldwide. Originally identified in epidemics associated with flooding in Asia, it nowadays shows very distinct genetic and epidemiological patterns. While HEV genotypes (HEV-) 1 and 2 are associated with the original outbreaks (waterborne diseases), HEV-3 and HEV-4 present a zoonotic pattern (associated with consumption of meat from infected animals), HEV-5 and 6 have been found only in wild boar in Japan, and HEV-7 and 8 have been detected in camels and dromedary seldom affecting humans. Brazil, with a precarious sanitary structure and being an important world meat producer, was the focus of this study in order to identify patterns of occurrence of HEV. After reviewing scientific studies, it was identified that the only genotype found in Brazil is HEV-3 and the area where there were more reports was the South region of the country. This is the region that produces more pork. These results indicate that HEV-3 is widespread in the country and sanitary surveillance is essential in the national production of pigs, as well as the implementation of monitoring protocols in hospitals.

**Abstract:**

Brazil is the fifth largest country in the world with diverse socioeconomic and sanitary conditions, also being the fourth largest pig producer in the world. The aim of the present systematic review was to collect and summarize all HEV published data from Brazil (from 1995 to October 2020) performed in humans, animals, and the environment, in a One Health perspective. A total of 2173 papers were retrieved from five search databases (LILACs, Mendeley, PubMed, Scopus, and Web of Science) resulting in 71 eligible papers after application of exclusion/inclusion criteria. Data shows that HEV genotype 3 (HEV-3) was the only retrieved genotype in humans, animals, and environment in Brazil. The South region showed the highest human seroprevalence and also the highest pig density and industry, suggesting a zoonotic link. HEV-1 and 2 were not detected in Brazil, despite the low sanitary conditions of some regions. From the present review we infer that HEV epidemiology in Brazil is similar to that of industrialized countries (only HEV-3, swine reservoirs, no waterborne transmission, no association with low sanitary conditions). Hence, we alert for the implementation of HEV surveillance systems in swine and for the consideration of HEV in the diagnostic routine of acute and chronic hepatitis in humans.

## 1. Introduction

In the last years, hepatitis E virus (HEV) has captured widespread attention when autochthonous hepatitis E cases started to be reported in industrialized countries [1]. Until then, hepatitis E was considered a rare disease in these countries and only associated with travelers returning from HEV endemic areas in Africa and Asia [2]. All the autochthonous cases reported in industrialized countries were caused by two HEV genotypes, namely HEV genotypes 3 (HEV-3) and 4 (HEV-4), that showed to have distinct epidemiological and clinical characteristics from the HEV genotype 1 (HEV-1) and HEV genotype 2 (HEV-2) circulating in developing countries. HEV-1 and HEV-2 are restricted to humans, transmitted by orofecal route through contaminated waters (usually linked to the lack of basic sanitation), and associated with large waterborne outbreaks of acute hepatitis in underdeveloped regions [3]. HEV-3 and HEV-4 are zoonotic viruses, common in domestic and wild pigs that infect humans as an accidental host through the consumption of uncooked contaminated pork products, being associated with sporadic human hepatitis cases [2,4]. Clinical features of these genotypes are also unique, with infections mostly asymptomatic in immunocompetent but with the capacity to progress to chronic hepatitis with liver cirrhosis in immunocompromised patients (such as organ transplant recipients and HIV patients), being also associated to diverse extra-hepatic manifestations (neurological and haematological) [2].

HEV is a non-enveloped positive-sense single-stranded RNA virus, belonging to *Hepeviridae* family, genera *Orthohepevirus*, species A, with eight genotypes currently recognized (HEV-1 to HEV-8) [3]. HEV-1 and HEV-4 have been detected in human cases, while HEV-5 and HEV-6 are genotypes strictly found in wild boar, HEV-7 and HEV-8 found in dromedary and Bactrian-camels [3]. There is only one report of HEV-7 in humans [5]. Currently, HEV-3 is subdivided into at least 11 subtypes (3a–3j, 3ra) [6].

Since swine are the main reservoir of HEV-3 as well as the main source of human infection and given that Brazil is the fourth largest pig producer in the world [7], a high HEV-3 circulation in the country is expected. Brazil is divided into 5 regions, namely North, Northeast, Midwest, Southeast and South, 26 states and a Federal District, with a total, of 5570 municipalities [8]. The South region has the highest pig production in the territory, accounting for 66.12% of the national production [7]. Moreover, Brazil is a country with continental dimensions, being the 5th largest country in the world with a population of circa 211 million, having a great extension of rural and urban areas with extremely diverse socioeconomic and sanitary conditions that influence infectious diseases dynamics [9]. There is today an increased awareness to monitor and survey the interfaces of human, animal, and environment in order to manage global health. Hence, the present systematic review aimed to collect and summarize all HEV published data from Brazil (from 1995 to October 2020) performed in humans, swine, other animals, and the environment, from a One Health perspective.

## 2. Materials and Methods

Exhaustive searches were carried out in the electronic databases: Latin American and Caribbean Health Sciences Literature (LILACs), Mendeley, PubMed, Scopus, and Web of Science. Two independent investigators (DFSDM and JRM) searched the databases, and included all studies published until October 2020. The study followed the protocol of the Preferred Reporting of Systematic Reviews and Meta-Analysis (PRISMA) [10], and the studies included should necessarily be published, indexed, and peer reviewed. No filters or other forms of search restrictions were used to achieve the greatest possible reach.

The literary search was made in the databases already mentioned above using the keywords (HEV OR Hepatitis E Virus) AND (Brazil). After reading the title and the abstract, papers that did not address Brazil as a scope or part of the scope, papers that did not study HEV, duplicate studies, review articles and experimental studies were excluded from this systematic review. Papers that did not make clear the information in the title and abstract were read in full and only those that contained the target content were included.

For the purpose of constructing this systematic review, all studies found in the databases that aimed at the parsing HEV in Brazil on their study scope were included, regardless of language, studied population or sample size. All authors independently screened the databases, and relevant information was extracted. Differences in opinions about whether to include an article were solved by consensus between all the authors.

## 3. Results

**Figure 1:**
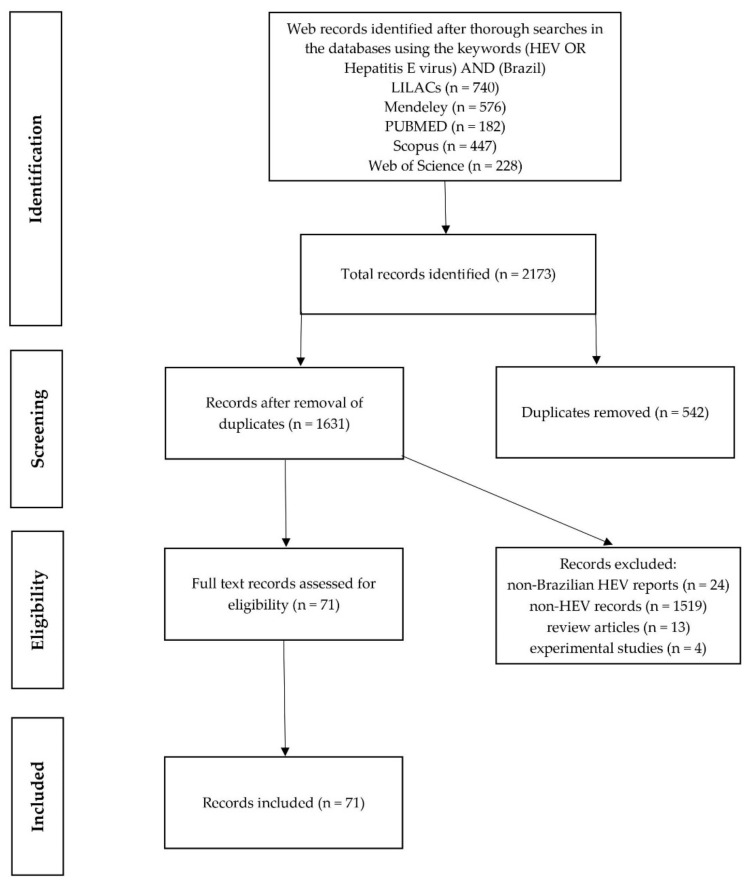
PRISMA Flow diagram showing the steps of the record selection procedure and reporting the strategies of inclusion/exclusion (explaining their reasons).

A total of 2173 papers were retrieved from the 5 databases used for the search (Figure 1). After removal of duplicated papers (*n* = 542), exclusion criteria were applied to eliminate non-related papers, namely papers classified as “non-Brazilian” (*n* = 24), “non-HEV” (*n* = 1519), as well as review articles and in vivo animal experimental studies.

**Figure 2:**
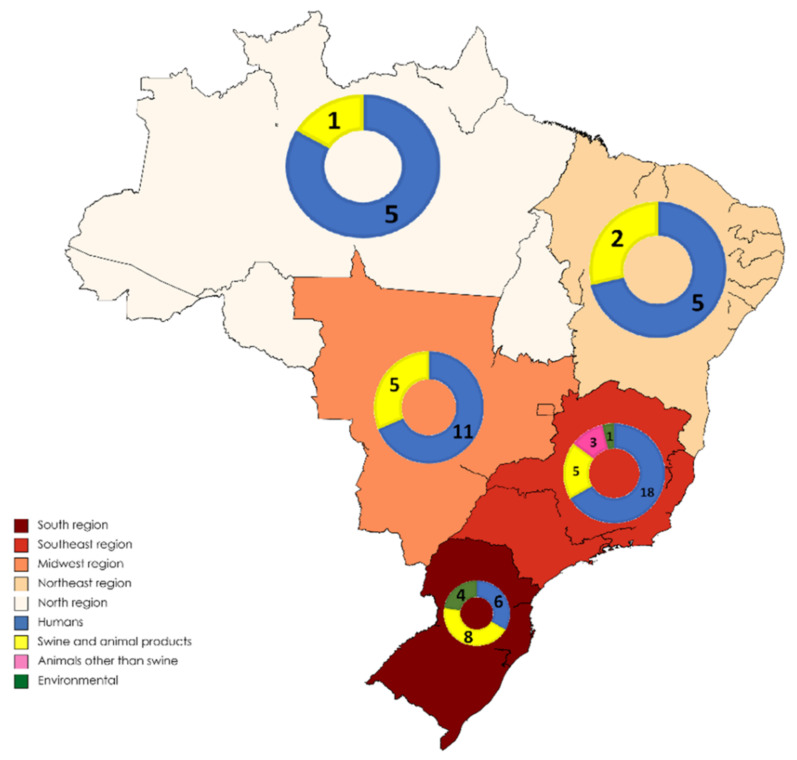
Distribution (number) of HEV studies according to the regions of Brazil and the origin (human, swine and animal products, animals other than swine, and environmental).

Application of inclusion and exclusion criteria generated a total of 71 eligible papers. They were all included in the study after being assessed by full-reading. The distribution of published papers by regions of Brazil and type of study can be observed in Figure 2. HEV studies in humans, swine and animal products, animals other than swine, and environment are summarized in Table 1, Table 2, Table 3 and Table 4, respectively.

### 3.1. HEV in Humans

HEV studies performed in humans in Brazil (Table 1) were focused on a variety of population groups and most were serological surveys.

Studies performed in populations from regions with lower sanitation and hygiene conditions in the North region found an anti-HEV IgG seroprevalence of 0.3% in afro descendants [14]. Studies done in poor communities in the Midwest region found an anti-HEV IgG seroprevalence of 3.3% and 10.66% in adults [28,30] and 4.5% in children [27]. In the Southeast region, a seroprevalence of 2.4% was found also in poor communities [34].

Seroprevalence studies focusing on rural settlements (Table 1) found anti-HEV IgG seroprevalences of 12.9% in the North [11], 3.4% [25], 3.9% [23], and 8.4% [26] in the Midwest, and 2.1% [35] and 20.7% [41] in the Southeast. Three of these studies performed in rural settlements were also focused on current and/or recent infections. The study of the Midwest region found 0% of anti-HEV IgM and HEV RNA [25] and the study of the North found 0.3% of anti-HEV IgM [14].

Several investigations were conducted in HIV patients from Brazil and found anti-HEV IgG seroprevalence of 4.1% [19] in the North, 0% [32], 6.7% [53] and 10.7% [42] in the Southeast. Anti-HEV IgM and HEV RNA in HIV patients was searched only in the Southeast region and found anti-HEV IgM in 0% [32], 0.83% [53], and 1.4% [42], while HEV RNA was detected in 2.23% [53] and 3.6% [32].

HEV studies in Brazil have also focused on transplant recipients (Table 1). Among those with kidney transplants, anti-HEV IgG seroprevalence was found to be of 2.5% [21] in the Midwest, and 3.1% [45] and 15% [43] in the Southeast. HEV RNA was found in 3.1% [45] and 10% [43] of kidney transplant recipients. Only two studies investigated HEV infection in liver transplant recipients, namely a case report in a pediatric patient [40] and a study in the Southeast region that found a seroprevalence of anti-HEV IgG and IgM of 8.1% and 2.6%, respectively [39].

Several investigations in Brazil were conducted in healthy blood donors and pregnant women (Table 1). Anti-HEV IgG seroprevalence in blood donors was found to be 0.45% [13] in the North, 2% [18] in the Northeast, 4% [26] in the Midwest, 4% [47], 4.3% [35] and 9.8% [37] in the Southeast, and 2.3% [50], 7.1% [53], 10% [54], 26% [49], and 40.25% [52] in the South. Of these studies, three also investigated current and/or recent infections by detecting anti-HEV IgM/HEV RNA, having found 0.33% and 0% [54], and 0.35% and 0.35% [53] respectiely, in the South. In the study of Southeast, anti-HEV IgM/RNA was 2.4% and 0%, but only IgG positive samples were tested [37].

Seroprevalence studies were also conducted in populations with occupational, exposure risk to HEV infection. In hospital employees anti-HEV IgG seroprevalences of 4.34% [48] and 5.9% [47] were found, while in recyclable waste pickers [24] and pig handlers [11] seroprevalences were 5.1% and 6.3%, respectively.

Molecular characterization of the HEV strains detected in humans in Brazil showed that all belonged to HEV-3 [32,33,40,45,53]. Further characterization of some of the strains identified subtypes 3b [33,40] and 3i [45].

### 3.2. HEV in Swine and in Animal Products

All studies performed in swine (Table 2) found evidence of HEV infection, either by using the detection of anti-HEV IgG and/or HEV RNA. Seroprevalence studies in younger pigs (<10 months) found an anti-HEV IgG prevalence of 8.6% in North region of Brazil [57] and 69.7% in the Midwest region [60]. The detection of HEV RNA in stools in this age group was 1.7% in the Northeast region [58] 7.94% in the North region [57] and 87.5% in Southwest [67].

In pigs from family-scale the anti-HEV IgG prevalence was 0% [61] and 67% [60] in the Midwest region, and 77.6% in the South region [71]. Regarding the detection of HEV RNA in stools of pigs from family-scale farms, 8% [61] and 24% [62] were found positive in the Midwest region, and 20% [68] in the South region.

Seroprevalence studies on slaughtered pigs showed anti-HEV IgG in 81.2% in Midwest [64] and 81.3% in the Northeast [59]. The detection of HEV RNA in bile from slaughtered pigs showed to be positive in 9.6% [66] and 15.2% [65] in Southeast and 0.84% in South [69].

The molecular characterization of the HEV found in pigs showed several subtypes (Table 2), namely 3b [62,66,68,69,70,71,72] 3c [57,65,71], 3d [61], 3f [57,58,62], 3h [61,71], and 3i [61,65].

Concerning wild boar, only two HEV seroprevalence studies were performed, both in the South region, having found a seroprevalence of 14.29% in Rio Grande do Sul state [73] while in Santa Catarina state, 1.55% [73] and 13.1% [74] seroprevalences were observed.

Regarding the HEV contamination of meat and meat products derived from swine and other animals (Table 2), HEV RNA was detected in 36% of the pig pâtés and blood sausages (morcilla) derived from pork [76]. In another study, no HEV was detected either in pig processed meats such as mortadella, sausage, salami, ham, and pate, or in the raw meat of bovine, swine, chicken, and capybara [75].

### 3.3. HEV in Animals Other Than Swine

None of the studies performed in free-living monkeys has found evidence of HEV infection, either by using the detection of anti-HEV IgG [11] or HEV RNA [77] (Table 3). Anti-HEV IgG was detected in cows (1.42%), dogs (6.97%), chickens (20%), and wild rodents (50%), but not in sheep and goats [11]. Two new viruses were detected in wild rodents, *Calomys* HEV (CaHEV) and *Necromys* HEV (NeHEV), and a new orthohepevirus species was proposed [78] (Table 3).

### 3.4. HEV in Environment

The detection of HEV RNA in waters (bathing/recreation waters, pig farm draining waters, settlement influenced waters), bivalve molluscs, and sediments was negative [55,76,79] (Table 4). In the two studies performed on pig slurry lagoons, HEV RNA was detected in 50% [66] and 100% [72] of the samples.

## 4. Discussion

The HEV studies in humans in Brazil started in the early 90s. The majority of these initial investigations were conducted in rural areas, possibly motivated by the HEV-1 and HEV-2 data from endemic regions in developing countries with similar poor sanitary conditions. The first HEV reports in Brazil focused on communities with low levels of sanitation, such as gold miners [29] and poor communities [28,30] from the Amazon area of the Midwest region, and from the Southeast region [34]. In these reports, the fecally contaminated water was pointed as a potential route of HEV transmission and the seroprevalences within these communities ranged from 0.45% in children to 10.66% in adults [27,28].

After the recognition of HEV-3 as being responsible for autochthonous hepatitis E in industrialized countries [81,82], HEV studies in Brazil started to focus on cases of acute non-A-C viral hepatitis in order to clarify the potential role of HEV in these undiagnosed cases [17,28,35], efforts that still motivate publications nowadays [15,36]. In general, markers of current and/or recent HEV infection (anti-IgM HEV and HEV RNA) have been detected but at a low prevalence, indicating that HEV was not the causal agent of the majority of these acute hepatitis cases.

Based on the knowledge that HEV-3 infection may progress to a chronic hepatitis in immunocompromised patients [3], some HEV studies in Brazil have focused on organ transplant recipients [39] and HIV patients [42]. In kidney transplants, HEV seroprevalence varied from infrequent (2.5%) [21] to frequent (15%) [43]. In liver transplant recipients the prevalence of anti-HEV antibodies showed to be higher than immunocompetent populations in Brazil, suggesting HEV infection as a possible cause of liver injury [39]. Concerning HIV patients, studies showed similar HEV seroprevalences when compared with blood donors indicating that HIV patients are not at risk for HEV infection [19,53].

Hepatitis E caused by HEV-1 and HEV-2 has been associated with morbidity and mortality in pregnant women [3]. Possibly motivated by this, some HEV seroprevalence studies have been performed in pregnant women in Brazil, however no risk for HEV seropositivity has been shown in this particular group when compared with the general population [13,35,49].

Several studies have evaluated the HEV seroprevalence in the general population of Brazil, with the majority using blood donors as the sampled group. A great range of HEV seroprevalence was observed, with the lowest detected in the North (0.45%) [13] and Northeast regions (2%) [18]. Mid-range levels of HEV seroprevalence were observed in the Midwest (4%) and Southeast (4%, 9.8%) regions [26,37,47]. In the South region, the five seroprevalence studies showed values of 2.3% [50], 7.1% [53], 10% [54], 26% [49], and 40.25% [52]. The high seroprevalence detected in the South has been justified for being the region in Brazil with the highest density of pig farms and the largest consumption of pig meat and related products [52]. In fact, pig breeding has been suggested to influence human HEV seroprevalence in other countries [83,84]. Epidemiologic surveys performed in rural population of Brazil, namely in the North [11] and in the Southeast regions, have found higher seroprevalences in these populations (12.9% and 20.7%, respectively) when compared to those previously reported on blood donors from the same regions [11,41]. This difference has been attributed to the lower sanitary conditions of the rural populations. Overall, the range of seroprevalences observed in Brazil has to be interpreted with caution since some studies were performed several decades apart and using different immunoassays. It is widely known that the different anti-HEV IgG immunoassays and their performance characteristics strongly influence HEV seroprevalence data [85].

Despite the strong evidence of widespread HEV circulation in Brazil, the recent report of the official governmental databases presented no notification of hepatitis E among the notified 216,379 hepatitis cases [86]. This draws attention to an underdiagnosis and/or underreporting of hepatitis E in Brazil. The underdiagnosing of hepatitis E cases has been reported elsewhere and is partly attributed to the fact that HEV testing has not been traditionally included in hepatitis differential diagnostic algorithms [87].

Many HEV studies in Brazil have focused on swine, which is understandable given the fact that this country is the 4th largest pig producer in the world, with more than 2 million breeders and producing 3975 thousand tons/year of pork meat, with the South region representing 66.12% of the national production [88]. Circulation of HEV in pigs of Brazil was observed either in large or family-scale herds, and in all age groups, based on HEV RNA presence in stools/biological fluids/organs (0.8–88.9%) or anti-HEV IgG seroprevalence (0–77.6%) [61,62,68,72]. Evidence for HEV infection in slaughtered pigs was also shown by the high seroprevalence (>80%) detected [59,64]. The circulation of HEV was also demonstrated in wild boars of Brazil with seroprevalences ranging from 1.55% to 14.29% [73,74]. HEV was inclusively found in pig pâtés and blood sausages derived from pork [76]. Overall, HEV is highly disseminated in the swine population throughout Brazil and might present a risk to animal handlers and pork consumers, mainly if pork meat and meat products are eaten raw or undercooked. The presence of HEV in pigs and derived pig products has been widely reported in other countries [84,88,89,90].

In the past years there has been an interest in studying HEV infection in non-human primates, inclusively *Macaca fascicularis* were used on experimental in vivo studies performed in Brazil to evaluate HEV pathogenesis [91,92,93]. HEV seroprevalences have been reported in farmed *Rhesus* monkeys in China (70.8%) [94] and in captive non-human primates in Italy (4.2%) [95] but the only seroprevalence study performed in Brazil in wild non-human primates did not detect any (0%) anti-HEV antibodies [11]. Furthermore, no HEV RNA was detected in the stools and livers of Golden-headed lion tamarins of Brazil [77].

Serological studies in Brazil also focused on other animals, having reported the presence of antibodies anti-HEV in cows, dogs, chicken, and wild rodents, but not in sheep and goats [11]. Antibodies against HEV have also been detected in dogs in the United Kingdom [96], in chicken, cows, wild rodents, sheep, and goats in China [97,98,99,100], chickens in Korea [101], sheep in Italy [102], but the zoonotic importance of these animals concerning HEV remain to be clarified. Noteworthy, two novel HEV strains were discovered in wild rodents from Brazil (*Calomys tener* and *Necromys asiurus*) [78].

Concerning the HEV studies that focused on the environment in Brazil, only water samples under the influence of swine farm effluents, namely slurry lagoons, were found positive for HEV [66,72]. Samples from the southern region of Brazil, with a high density of swine production, detected HEV in up to 100% of the samples analyzed [72]. This same region coincides with the highest rates of human seropositivity for HEV and is also the region with the highest concentration of pig production in the country. This fact, analyzed from the One Health perspective, highlights the zoonotic character of this virus. Swine-influenced waters contaminated by HEV have been frequently detected and reported in other countries [103,104]. In the studies of Brazil, HEV was not detected in bivalve molluscs, recreation waters, or even in waters that drained effluents from pig farms or waters of poor quality, very close to human settlements [76,79,80]. However, studies in other countries have reported HEV in bivalve molluscs [105,106,107], seawater [108], and wastewater [109,110]. These discrepancies of detection of HEV in environment samples could be in part due to the low concentration of HEV and complexity of the matrices, two well-known limiting factors of the detection of enteric viruses in environmental samples.

Concerning the molecular characterization of HEV strains detected in Brazil, studies showed that all HEVs found in Brazil were classified as HEV-3 (6 studies in humans, 15 in swine and animal products, and 2 on environmental samples). HEV-3 is known to have a zoonotic (swine) origin and the subtypes 3b and 3i were detected in humans [33,40,45] and pigs [61,62,65,66,68,69,70,71,72], while the subtypes 3c [57,65,71], subtype 3d [61], subtype 3f [57,58,62] and subtypes 3h [61,71] have been only detected in pigs. As molecular studies have been performed using several molecular assays and primer choices, different regions of HEV have been targeted and characterized. This clearly hampers the robust classification of HEV subtypes and, consequently, a solid comparison between subtypes, hence caution must be taken when analyzing this data. In fact, attention should be paid to several factors that could bias the interpretation of results here presented. A clear focus has been given to human samples with little attention to animal or environmental matrices, most likely due to the initial understanding of this disease, not known to be zoonotic at that time. Additionally, not only a higher number of studies have also focused on the South where the highest density of pig farms is present but also a vast diversity of sample sizes has been used throughout the studies, making it difficult to robustly compare results. Further studies spatially dispersed are for these reasons recommended.

The present systematic review is not the first that targets HEV in Brazil. The two published so far have centered only on human infection [111,112] while here we present for the first time a perspective focusing on the One Health triad, having included HEV studies on humans, animals, and environment. A One Health approach makes it possible to look at issues such as zoonotic diseases, food safety, and food security, as well as environmental contamination and other aspects. In this perspective this review evidenced that the scientific community has approached the topic of HEV on every aspect of environment, human, and animal systems individually, however when compiled, this translates into data that broadens the scope to One Health.

## 5. Conclusions

Overall, this systematic review shows that HEV-3 was the only retrieved genotype in humans, animals, and environment in Brazil. The South region showed the highest HEV seroprevalence in humans, which curiously is also the region with the highest pig density, swine industry, and pig HEV circulation, suggesting a zoonotic link. HEV- 1 and HEV-2 were not detected in any of the studies performed in Brazil, even in those focusing on low sanitary condition communities. This allowed us to infer that HEV epidemiology in Brazil is similar to that of industrialized countries (only HEV-3 circulation, swine reservoirs, no waterborne transmission, no association with low sanitary conditions). Hence, we alert for the implementation of HEV surveillance systems in swine and for the inclusion of HEV in the diagnostic routine of acute and chronic hepatitis in humans. More sequence data are needed on HEV strains circulating in humans, animals, and the environment to further evidence the zoonotic origin of HEV infection in Brazil.

## Figures and Tables

**Table 1:** HEV in humans, Brazil.

Region of Brazil	Sampling Location	Sampling Date	Population Details	Type of Samples	Hev Diagnostic Assay	Number of Positive/Total (%)	Hev Genotype	Additional Data	Reference
North	Acre	2004	Rural settlements	Sera	IgG/IgM (only on IgG positive cases) (EIA ^1,2^ + immunoblot ^4^)	IgG 50/388 (12.9%), IgM 7/43 (16.3%)	-	The odds for HEV seropositivity increased by 3.3% for each additional year of age	[11]
	Acre	1997	Riverine communities of amazon basin	Sera	IgG (EIA ^3^)	14/349 (4%)	-	-	[12]
	Amazonas	-	Blood donors, hemodialyzed, pregnant women	Sera	IgG (EIA ^3^)	Blood donors 1/227 (0.45%), hemodialyzed 1/192 (0.52%), pregnant women 0/100 (0%)	-	-	[13]
	Pará	2015	Rural afro-descendant communities	Sera	IgM/IgG (EIA ^2^ + immunoblot ^4^), RNA (RT-qPCR)	IgM 2/535 (0.3%), IgG 2/535 (0.3%), RNA 0/9 (0%)	-	Afro-descendant rural communities from the eastern Brazilian Amazon had low HEV infection	[14]
	Pará	1993–2014	Non-A-C hepatitis or suspected cases of HEV infection	Sera	IgM/IgG (EIA ^2^ + immunoblot ^4^), RNA (RT-qPCR)	IgM 11/318 (3.4%), IgG 19/318 (5.9%), RNA 0/318 (0%)	-	HEV low circulation rate even between suspected cases in the Eastern Brazilian Amazon	[15]
Northeast	Bahia	1995–1999	Acute hepatitis cases	Sera	IgG/IgM (only on IgG positive cases) (EIA ^3,5^)	Anti-HEV in hepatitis A cases: IgG 15/40 (38%), IgM 4/15 (26.67%); anti-HEV in hepatitis B cases: IgG 4/42 (10%), IgM 0/4 (0%); anti-HEV in hepatitis non-A-C: IgG 2/12 (8.34%), IgM 1/2 (50%)	-	IgG prevalence was significantly higher in patients with hepatitis A (38%) compared to the hepatitis B group (10%) (*p* < 0.01)	[16]
	Bahia	1992–1996	Acute hepatitis cases	Sera	IgM/IgG (EIA ^3^)	IgM 0/43 (0%), IgG 5/43 (12%)	-	-	[17]
	Bahia	1992–1994	Blood donors, hemodialyzed, acute viral hepatitis, schistosomiasis cases	Sera	IgG (EIA ^3^)	Blood donors 4/200 (2%), hemodialyzed 0/392 (0%), acute viral hepatitis 14/79 (17.7%), schistosomiasis 3/30 (10%)	-	Among acute viral hepatitis cases, those with hepatitis A had a higher frequency of positivity compared with all other hepatotropic viruses (*p* < 0.003)	[18]
	Pernambuco	2016–2017	HIV patients	Sera	IgG (EIA ^2^), RNA (RT-PCR)	IgG 15/366 (4.1%), RNA 0/366 (0%)	-	Several risk factors were evaluated: age, years of school, sexual orientation, oral-anal sex, use of injectable drugs and piped water availability. Piped water showed to be a protective factor for HEV infection (*p* = 0.018)	[19]
	Pernambuco	-	Schistosomiasis cases	Sera	IgM/IgG (EIA ^6^), RNA (RT-qPCR)	IgM 0/80 (0%), IgG 15/80 (18.8%), RNA 0/80 (0%)	-	-	[20]
Midwest	Goiás	2014	Renal transplant recipients	Sera	IgM/IgG (EIA ^2^), RNA (RT-qPCR)	IgM 0/316 (0%), IgG 8/316 (2.5%), RNA 0/316 (0%)	-	HEV infection was infrequent in kidney transplant recipients in Central Brazil	[21]
	Goiás	2012–2014	Non-A-C hepatitis cases	Sera	IgM/IgG (EIA ^2^ + immunoblot ^4^), RNA (RT-qPCR)	IgM 1/379 (0.3%), IgG 20/379 (5.3%), RNA 0/379 (0%)		Sociodemographic characteristics were evaluated: sex, age, marital status, ethnicity, schooling and monthly income. Low education level (*p* = 0.005) and living in rural areas (*p* = 0.056) were found to be associated with HEV seropositivity	[22]
	Goiás and Mato Grosso do Sul	2011–2012	Rural settlements	Sera	IgM/IgG (EIA ^2^)	Anti-HEV (total) 36/923 (3.9%)	-	-	[23]
	Goiás	2010–2011	Recyclable waste pickers	Sera	IgM/IgG (EIA ^2^ + immunoblot ^4^), RNA (Nested RT-PCR)	IgM 3/431 (0.7%), IgG 22/431 (5.1%), RNA 0/3 (0%)	-	Sociodemographic characteristics were evaluated: sex, age, marital status, ethnicity, schooling and monthly income. Age > 40 years wsa found to be associated (*p* < 0.01) with HEV seropositivity	[24]
	Goiás	2011	Rural settlements	Sera	IgM/IgG (EIA ^2^ + immunoblot ^4^), RNA (RT-qPCR)	IgM 0/464, IgG 16/464 (3.4%), RNA 0/464 (0%)	-	Sociodemographic characteristics were evaluated: sex, age, marital status, ethnicity, schooling and monthly income. Dwelling in a rural settlement for >5 years was associated (*p* = 0.025) with HEV seropositivity	[25]
	Mato Grosso	2009–2010	Blood donors, rural settlements	Sera	IgG (EIA ^7^)	Blood donors 4/101 (4%), rural settlements 26/310 (8.4%)	-	Living in rural settlements was not found to be a risk factor for HEV infection (*p* = 0.206)	[26]
	Mato Grosso	1998	Children (3–9 years old)	Sera	IgG (EIA ^3^)	3 years 0/8 (0%), 4 years 0/13 (0%), 5 years 5/48 (10.4%), 6 years 5/87 (5.7%), 7 years 1/106 (0.9%), 8 years 8/124 (6.4%), 9 years 3/101 (3%)	-	The overall HEV seroprevalence in children (3–9 years old) was 4.5%	[27]
	Mato Grosso	1995	Community of Amazon non-A-C acute hepatitis and asymptomatic cases	Sera	IgM/IgG (EIA ^3^)	Non-A-C 2/16 (12.5%), asymptomatic 7/66 (10.60%)	-	Authors claim to be the first study reporting evidence for HEV infection in brazilian Amazon	[28]
	Mato Grosso	1993	Gold miners	Sera	IgG (EIA ^5^)	6/97 (6.18%)	-	Authors claim to be the first HEV survey in Brazil	[29]
	Mato Grosso	-	Amazon poor community	Sera	IgG (EIA ^3^)	10/299 (3.3%)	-	-	[30]
	Mato Grosso do Sul	2013–2015	Crack cocaine users	Sera	IgG/IgM (EIA ^6^), RNA (RT-qPCR)	IgM 2/698 (0.28%), IgG 99/698 (14.2%), RNA 0/2 (0%)	-	-	[31]
Southeast	Rio de Janeiro	2012–2014	HIV positive	Sera	IgM/IgG (EIA ^2^), RNA (RT-qPCR)	IgM 0/280 (0%), IgG 0/280 (0%), RNA 11/280 (3.6%)	3	The RNA load ranged from 10^2^–10^8^ copies/mL	[32]
	Rio de Janeiro	2004–2008	Non-A-C hepatitis	Sera	IgM/IgG (EIA ^1^), RNA (RT-qPCR)	IgM 1/64 (1.56%), IgG 1/64 (1.56%), HEV RNA 1/64 (1.56%)	3b	Authors claim to be the first report of an autochthonous HEV infection in Brazil. A single sample tested positive for both IgM/IgG and HEV-RNA (viral load of 10^5^ copies/mL)	[33]
	Rio de Janeiro	1999	Poor community	Sera	IgG (EIA ^3^)	17/699 (2.4%)	-	-	[34]
	Rio de Janeiro	1994–1998	Blood donors, pregnant women, non-A-C hepatitis cases, hemodialyzed, intravenous drug users (IVDU), individuals living in the rural and urban areas	Sera	IgG (EIA ^3^)	Blood donors 4/93 (4.3%), pregnant women 3/304 (1%), non-A-C 3/146 (2.1%), hemodialyzed 4/65 (6.2%), IVDU 12/102 (11.8%), rural area 3/145 (2.1%), urban area 0/260 (0%)	-	-	[35]
	Rio de Janeiro	-	Pig handlers	Sera	IgG (EIA ^8a^)	2/32 (6.3%)	-	-	[11]
	São Paulo	2015–2016	Chronic hepatitis C cases	Sera	IgG/IgM (only on IgG positive and inconclusive cases) (EIA ^6^)	IgG 63/618 (10.2%), IgM 0/66 (0%)	-	HEV seroprevalence in patients with cirrhosis was significantly higher than in patients without cirrhosis (13.2% vs 8%, *p* = 0.04)	[36]
	São Paulo	2014	Blood donors	Sera	IgG/IgM (only on IgG positive cases) (EIA ^6^), RNA (RT-qPCR)	IgG 49/500 (9.8%), IgM 1/49 (2.04%), RNA 0/49 (0%)	-	-	[37]
	São Paulo	2013	Transfusion-dependent thalassemia or sickle cell disease (SCD)	Sera	IgG (EIA ^6^), RNA (RT-PCR)	IgG: Thalassemia 8/40 (20%), SCD 4/52 (7.7%); RNA 0/92 (0%)	-	The overall anti-HEV IgG seroprevalence in patients with thalassemia and SCD was 13.0%	[38]
	São Paulo	2013	Liver transplant recipients	Sera	IgM/IgG (EIA ^2^)	IgM 6/284 (2.6%), IgG 23/284 (8.1%)	-	-	[39]
	São Paulo	2012	Pediatric liver transplant case	Sera	IgM/IgG (EIA ^2^), RNA (RT-qPCR)	IgM(+), IgG (+), RNA (+)	3b	Authors claim to be the first report of chronic and/or pediatric HEV infection in Latin America. RNA showed a load of 4.5 log_10_ copies/mL	[40]
	São Paulo	2011–2013	Urban and rural residents	Sera	IgM/IgG (EIA ^2,6^ + immunoblot ^7^,) RNA (RT-qPCR)	IgG 50/242 (20.7%), RNA 0/244	-	-	[41]
	São Paulo	2007–2013	HIV positive	Sera	IgM/IgG (EIA ^2^ + immunoblot ^4^), RNA (RT-qPCR)	IgM 5/354 (1.4%), IgG 38/354 (10.7%), RNA 0/354 (0%)	-	-	[42]
	São Paulo	2001–2011	Renal transplant recipients	Sera	IgG (EIA ^2^), RNA (Nested RT-PCR)	IgG 28/192 (15%), RNA 20/192 (10%)	-	Exposure to HEV during hemodialysis suggested as the cause of the high prevalence	[43]
	São Paulo	1998–2013	Non-A-C hepatitis cases	Sera	IgM/IgG (EIA ^9^)	IgM (from 2006 to 2013) 27/552 (4.1%), IgG (from 1998 to 2013) 47/2.271 (2.1%)	-	The highest IgM/IgG seroprevalences were observed in latest years, namely 2011 to 2013: IgM (8.8% in 2011, 5.8% in 2012, 7.4% in 2013); IgG (5.9% in 2011, 8.6% in 2012, 6.1% in 2013)	[44]
	São Paulo	1998–2007	Renal transplant recipients	Sera	IgG (EIA ^2^), RNA (Nested RT-PCR)	IgG 0/96 (0%), RNA 3/96 (3.1%)	3i	Authors claim to be the first report of HEV infection with subtype 3i in Brazil	[45]
	São Paulo	-	Blood donors	Sera	HEV-specific T-cell, RNA (RT-PCR)	T-cell response 570/33,582 (1.7%), RNA 4/29 (13.79%)	-	-	[46]
	São Paulo	-	Group I (Blood donors) A: normal ALT levels; B: high ALT levels; Group II (Women test for HIV) C: prostitutes; D: non-prostitutes; Group III (hospital employees) E; care workers; F: cleaning service workers	Sera	IgG (EIA ^3^)	Group I 8/205 (4%): A 5/165 (3%), B 3/40 (7.5%). Group II 38/214 (17.7%): C 3/21 (14.2%), D 35/193 (18.1%). Group III 10/170 (5.9%): E 3/117 (2.6%), F 7/53 (13.2%)	-	-	[47]
	São Paulo	-	Hospital settings, hemodialyzed	Sera	IgG (EIA ^3^)	Hospital settings 1/23 (4.34%), hemodialyzed 2/38 (5.26%)	-	The overall anti-HEV IgG seroprevalence was 4.9%	[48]
South	Paraná	2002–2003	Pregnant women, female blood donors	Sera	IgG (EIA ^6^), RNA (Nested RT-PCR)	IgG: Pregnant women 40/209 (19%), female blood donors 51/199 (26%); RNA 0/408 (0%)	-	The overall IgG positivity of pregnant women and female blood donors was 22.5%. No significant difference (*p*= 0.11) in the HEV seroprevalence was observed between the two groups	[49]
	Paraná	1999	Blood donors	Sera	IgG (EIA ^3^)	23/996 (2.3%)	-	-	[50]
	Paraná	-	Young patient with neurological disorders	Sera	IgM/IgG, RNA (Nested RT-PCR)	IgM (+), IgG (+), RNA (+)	3	Case report about young patient with severe chronic hepatitis and presenting Epstein-Barr virus (EBV) in their cerebrospinal fluid	[51]
	Rio Grande do Sul	2015	Blood donors	Sera	IgG (EIA ^8b^)	314/780 (40.25%)	-	An *in house* ELISA with 91.4% sensitivity and 95.9% specificity was developed and used	[52]
	Rio Grande do Sul	2012–2015	Blood donors, HIV positive	Sera	IgM/IgG (EIA ^2^ + immunoblot ^4^), RNA (RT-qPCR)	Blood donors: IgM 1/281 (0.35%), IgG 20/281 (7.1%), RNA 1/281 (0.35%); HIV positive: IgM 3/360 (0.83%), IgG 24/360 (6.7%), RNA 8/360 (2.23%)	3	The RNA load ranged from 2500–4000 copies/mL	[53]
	Santa Catarina	2014	Blood donors	Sera	IgM/IgG (EIA ^6^), RNA (RT-qPCR)	IgM 1/300 (0.33%), IgG 30/300 (10%), RNA 0/300 (0%)	-	-	[54]
Brazil (nationwide)		2014–2018	Viral hepatitis cases	Sera	HEV assays not defined	0/216,397 (0%)	-	Data compiled from official national notifications	[55]
		2010–2012	Children with acute flaccid paralysis or Guillain-Barré syndrome	Stools	RNA (RT-qPCR)	0/325 (0%)	-	HEV infection could not be associated with the neurological disorders	[56]

^1^ bioELISA^®^ HEV IgG/IgM (Biokit™, Barcelona, Spain); ^2^ *recom*Well^®^ HEV IgM/*recom*Well^®^ HEV IgG (Mikrogen, Diagnostik, Munich, Germany); ^3^ IgG Abbott Diagnostika™(Wiesbaden, Germany); ^4^ *recom*Line^®^ HEV IgG/IgM (Mikrogen, Diagnostik, Munich, Germany); ^5^ GLD HEV (Genelabs Diagnostics^®^, Singapore, Singapore); ^6^ Wantai™ HEV-IgG ELISA kit (Wantai Biological, Beijing, China); ^7^ MPD^®^ HEV ELISA (MP Diagnostics™, MP Biomedicals, CA, USA); ^8^ in-house: ^a^ two HEV recombinant proteins, a mosaic protein (MP-II) and a protein containing region 452–617 aa of the ORF2 of the HEV Burma strain were used as coating antigens; ^b^ ORF2 recombinant protein was used as coating antigen; ^9^ Hepatitis E Virus (HEV) Antibody (IgG) Quest Diagnostics^®^ (New York, NY, USA);

**Table 2:** HEV in swine and animal products, Brazil.

Region of Brazil	Sampling Location	Sampling Date	Animal & Production Details	Type of Samples	HEV Diagnostic Assay	Number of Positive Samples/Total Tested (%)	HEV Genotype	Additional Data	References
HEV in swine									
North	Pará	2010	Slaughtered (6 months old)	Sera, livers, stools	IgM/IgG (ELISA ^1^ + immunoblot ^2^, RNA (Nested RT-PCR)	IgM 0/151 (0%), IgG 13/151 (8.6%); RNA: serum 4/151 (2.64%), livers 6/151 (3.97%), stools 12/151 (7.94%)	3c, 3f	The global rate of HEV infection was 9.9%. Coinfection with two subtypes was observed in one pig	[57]
Northeast	Pernambuco	2017	From intensive/semi-intensive herd systems (2–6 months old)	Stools	RNA (RT-PCR)	2/119 (1.7%)	3f	-	[58]
	Pernambuco	-	Slaughtered, intensive/semi- intensive herd systems	Sera	IgG (EIA ^3^)	Slaughtered 78/96 (81.3%), herds 188/229 (82.1%)	-	Not performing disinfection (after cleaning) and mixed drinking water (stagnant and running) were risk factors for IgG prevalence while semi-intensive production system had a protective effect	[59]
Midwest	Federal District	2014	Young (6–10 months old) and adults (11–48 months old) from 234 family herds	Sera	IgG (EIA ^3^)	Young 85/122 (69.7%), adults 219/327 (67.0%)	-	No difference was observed in IgG seropositivity by gender or age	[60]
	Mato Grosso	2015	Family-scale herds	Sera, stools	RNA (Nested RT-PCR)	Sera 0/150 (0%), stools 12/150 (8%)	3d, 3i, 3h	From the 15 herds tested, 8 (53.3%) had pigs infected with HEV	[61]
	Mato Grosso	-	Large and family scale herds	Livers, gallbladder, small & large intestines, bile, stools	HEV antigen (Immunohistochemistry with polyclonal primary antibody-4), RNA (Nested RT-PCR)	Large-scale herds/RNA and HEV antigen: livers 0/25 (0%), bile 0/25 (0%), stools 0/25 (0%). Family scale/RNA: livers 6/25 (24%), bile 7/25 (28%), stools 6/25 (24%). Family scale/HEV antigen: livers 1/25 (0.25%), small intestine 7/25 (28%), large intestine 4/25 (16%)	3b, 3f	HEV was not detected in pigs from large-scale farms, only in family herds	[62]
	Mato Grosso	-	Piglets (from IgG positive sows)	Sera	IgG (EIA^5a^)	8/47 (17%)	3	Piglets were monitored after weaning and seroconversion (due to natural infection) was observed in 17% of 6–8 weeks old. Genotyping was performed in a stool pool (from piglets 10–12 weeks old)	[63]
	Mato Grosso	2002–2003	Slaughtered (28 weeks old)	Sera	IgG (EIA ^5a^)	211/260 (81.2%)	-	-	[64]
Southeast	Minas Gerais	2012	Slaughtered	Bile	RNA (RT-qPCR)	51/335 (15.2%)	3c, 3i	Authors suggest intragenotype HEV recombination	[65]
	Rio de Janeiro	2008	Slaughtered	Bile	RNA (RT-qPCR)	11/115 (9.6%)	3b	Viral loads varied from 10^1^–10^5^ copies/mL	[66]
	Rio de Janeiro	-	Piglets (from IgG positive sows)	Sera	IgM/IgG (EIA ^5a^), RNA (Nested RT-PCR)	Sera (16 weeks old): IgM 1/26 (3.84%), IgG 0/26 (0%); sera (22 weeks old): IgM 0/26 (0%), IgG 23/26 (88.4%); sera (13 weeks old): RNA 8/26 (30.76%)	3	Piglets were monitored after weaning and seroconversion (due to natural infection) was observed in 88.4% of 22 weeks old. Transferred antibodies from colostrum were observed in 92.3% piglets, decreasing weekly until 16 week-old	[63]
	Rio de Janeiro	-	Two large herds, A and B (age range 1 to >25 weeks old in B)	Sera	IgG (EIA ^5a^)	Herd A 17/70 (24.3%), herd B 227/357 (63.7%)	-	-	[11]
	São Paulo	-	Young (40–60 days old)	Stools	RNA (RT-PCR)	7/8 (87.5%)	3	-	[67]
South	Paraná	2014	Family scale herds (22 weeks old)	Stools	RNA (Nested RT-PCR/RT-qPCR)	34/170 (20%)	3b	Among the 34 positive samples, only 4 (11.8%) presented viral loads higher than 10^3^ copies/mL	[68]
	Paraná	2010	Slaughtered	Liver, bile	RNA (Nested RT-PCR)	Liver 2/118 (1.7%), bile 1/118 (0.84%)	3b	-	[69]
	Paraná	2009	Herds with animals of different ages	Stools	RNA (Nested RT-PCR)	1–4-week-old 2/25 (8%), 5–8 weeks old 1/33 (3%), 9–24 weeks old 26/170 (15.3%), >1-year-old 3/99 (3%)	3b	-	[70]
	Rio Grande do Sul	2012–2014	Family-scale herds	Sera	IgG (EIA ^5b^), RNA (Nested RT-PCR)	IgG (2012) 567/731 (77.6%), IgG (2014) 467/713 (65.5%), RNA (2014) 6/713 (0.8%)	3b, 3c, 3h	-	[71]
	Rio Grande do Sul	-	Large-scale herds	Stools	RNA (Nested RT-PCR)	8/9 (88.9%)	3b	-	[72]
	Rio Grande do Sul	2012–2016	Family scale pig herds and wild boars	Sera	Antibodies (EIA ^3^)	Pigs 139/261 (53.26%), wild boar 8/56 (14.29%)	-	This study shows pigs from family scale can play a more important role as a HEV reservoirs than wild boars (*p* < 0.001)	[73]
	Santa Catarina	2017–2018	Wild boars	Sera	Antibodies (EIA ^3^)	8/61 (13.1%)	-	-	[74]
	Santa Catarina	2012–2016	Family scale pig herds and wild boars	Sera	Antibodies (EIA ^3^)	Pigs 39/121 (32.23%), wild boar 3/193 (1.55%)	-	This study shows pigs from family scale can play a more important role as a HEV reservoirs than wild boars (*p* < 0.001)	[73]
HEV in animal products									
South	Rio Grande do Sul	2015–2016	Edible products of animal origin	Bovine, swine, chicken and capybara raw meats, processed meats (mortadella, sausage, salami, ham, pâté)	RNA (Nested RT-PCR)	Bovine 0/57 (0%), swine 0/30 (0%), chicken 0/29 (0%), capybara 0/1 (0%), mortadella 0/8 (0%), sausage 0/12 (0%), salami 0/14 (0%), ham 0/4 (0%), pâté 0/4 (0%)	-	-	[75]
	Rio Grande do Sul	2015	Pork products	Pâtés, blood sausage (*morcilla*)	RNA (Nested RT-PCR)	18/50 (36%)	3	-	[76]

^1^*recom*Well^®^ HEV IgM/ *recom*Well^®^ HEV IgG (Mikrogen, Diagnostik, Munich, Germany); ^2^ *recom*Line^®^ HEV IgG/IgM (Mikrogen, Diagnostik, Munich, Germany); ^3^ PrioCHECK™ AB HEV antibody ELISA kit (Thermo Fisher, Zurich, Switzerland); ^4^ HEV antibody (Abbiotec™, California, USA); ^5^ in-house: ^a^ two HEV recombinant proteins, a mosaic protein (MP-II) and a protein containing region 452–617 aa of the ORF2 of the HEV Burma strain used as coating antigens; ^b^ in-house indirect ELISA containing recombinant HEV-ORF2p antigen.

**Table 3:** HEV in animals other than swine, Brazil.

Region of Brazil	Sampling Location	Sampling Date	Animal Species	Type of Samples	HEV Diagnostic Assay	Number of Positive Samples/Total Tested (%)	HEV Genotype	Additional Data	Reference
Southeast	Rio de Janeiro	2012–2016	Golden-headed lion tamarin (*Leontopithecus chrysomelas*)	Stools, livers	RNA (RT-PCR)	stools 0/101 (0%), livers 0/95 (0%)	-	-	[77]
	Rio de Janeiro	-	Captive New World monkeys *(Callithrix jacchus, C. kuhli, C. asiurus, C. penicilata, C. argenta, Aotus* sp.), dogs, cows, sheeps, goats, chickens, wild rodents (*Nectomus* sp.)	Sera	IgG (EIA ^1^)	Monkeys 0/42 (0%), dogs 3/43 (6.97%), cows 1/70 (1.42%), sheeps 0/12 (0%), goats 0/5 (0%), chickens 5/25 (20%), wild rodents 2/4 (50%)	-	-	[11]
	São Paulo	2008–2013	Wild rodents (*Akodon montensis, Calomys tener, Oligoryzomys nigripes, Necromys asiurus, Mus musculus*)	Sera	RNA (RT-PCR)	*A. montensis* 0/199 (0%), *C. tener* 4/109 (3.66%), *O. nigripes* 0/63 (0%), *N. asiurus* 3/252 (1.19%), *M. musculus* 0/24 (0%)	A new orthohepevirus species was proposed	Novel strains were termed *Calomys* HEV (CaHEV) and *Necromys* HEV (NeHEV)	[78]

^1^ in-house, two HEV recombinant proteins, a mosaic protein (MP-II) and a protein containing region 452–617 aa of the ORF2 of the HEV Burma strain were used as coating antigens

**Table 4:** HEV in environmental samples, Brazil.

Region of Brazil	Sampling Location	Sampling Date	Matrices	Detection Method (RNA)	Number of Positive Samples/Total Tested (%)	HEV Genotype	Additional Data	References
South	Rio Grande do Sul— Vale do Taquari	2016–2017	Water	Nested RT-PCR	0/32 (0%)	-	Samples were from area that drains effluents from numerous pig farms	[79]
	Rio Grande do Sul—Northern coast	2016–2017	Water and bivalves (*Donax hanleyanus*)	Nested RT-PCR	Water 0/42 (0%), bivalves 0/42 (0%)	-	Samples were from recreation beaches	[80]
	Rio Grande do Sul—Sinos river	2012–2014	Water and sediment	Nested RT-PCR	Water 0/250 (0%), sediment 0/68 (0%)	-	Sampling site had poor water quality, very close to human settlements	[76]
	Rio Grande do Sul—Vale do Taquari	-	Swine slurry lagoon water	Nested RT-PCR	8/8 (100%)	3b	Samples were from one of the largest swine producers in Rio Grande do Sul and a great public initiative for decontamination of water bodies was initiated at the time of this study	[72]
Southeast	Rio de Janeiro—North and hill region	2008	Swine slaughterhouse effluent	RT-qPCR	3/6 (50%)	3b	RNA was found with mean viral load of 10^2^ genome copies/mL in effluent	[66]

## Data Availability

Not applicable.
